# Multi-target regulation of intestinal microbiota by berberine to improve type 2 diabetes mellitus

**DOI:** 10.3389/fendo.2022.1074348

**Published:** 2022-11-18

**Authors:** Qiongyao He, Hui Dong, Yujin Guo, Minmin Gong, Qingsong Xia, Fuer Lu, Dingkun Wang

**Affiliations:** ^1^ Institute of Integrated Traditional Chinese and Western Medicine, Tongji Hospital, Tongji Medical College, Huazhong University of Science and Technology, Wuhan, Hubei, China; ^2^ Department of Integrated Traditional Chinese and Western Medicine, Tongji Medical College, Tongji Hospital, Huazhong University of Science and Technology, Wuhan, Hubei, China

**Keywords:** BBR, T2DM, intestinal microbiota, microbial metabolites, metabolic diseases

## Abstract

Type 2 diabetes mellitus (T2DM) and its complications are major public health problems that seriously affect the quality of human life. The modification of intestinal microbiota has been widely recognized for the management of diabetes. The relationship between T2DM, intestinal microbiota, and active ingredient berberine (BBR) in intestinal microbiota was reviewed in this paper. First of all, the richness and functional changes of intestinal microbiota disrupt the intestinal environment through the destruction of the intestinal barrier and fermentation/degradation of pathogenic/protective metabolites, targeting the liver, pancreas, visceral adipose tissue (VAT), etc., to affect intestinal health, blood glucose, and lipids, insulin resistance and inflammation. Then, we focus on BBR, which protects the composition of intestinal microbiota, the changes of intestinal metabolites, and immune regulation disorder of the intestinal environment as the therapeutic mechanism as well as its current clinical trials. Further research can analyze the mechanism network of BBR to exert its therapeutic effect according to its multi-target compound action, to provide a theoretical basis for the use of different phytochemical components alone or in combination to prevent and treat T2DM or other metabolic diseases by regulating intestinal microbiota.

## Graphical abstract

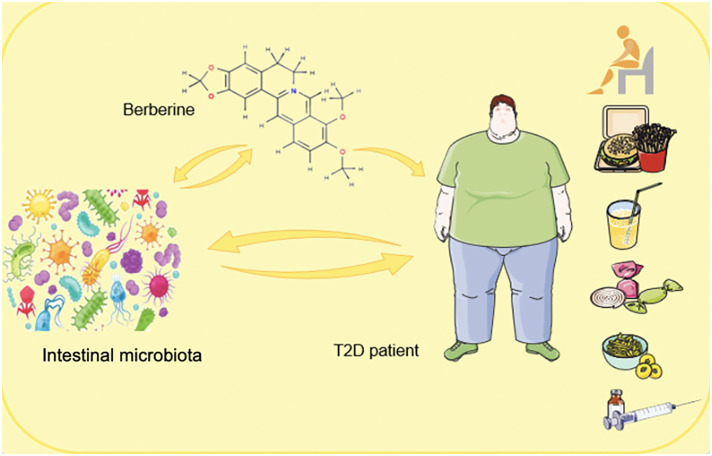

## 1 Introduction

Diabetes mellitus (DM) is a group of metabolic diseases characterized by hyperglycemia, caused by deficiencies in insulin secretion and/or insulin action (1). In the past few decades, great changes have taken place in human lifestyles around the world. Reducing the level of physical activity and increasing animal food consumption and dietary fat intake make the incidence of T2DM more favorable (2–4). There were about 537 million adults with DM worldwide in 2021 and it is expected to reach 784 million by 2045 (5). DM has brought profound psychological and physical troubles to patients and is related to several serious complications, which have aroused great concern all over the world (6, 7).

The human intestinal tract is a bioreactor with microbiota, containing hundreds or thousands of bacterial groups (8). Most bacteria belong to six well-known bacteria/phyla: Phaeophyta, Bacteroides, Proteus, Actinomycetes, Clostridium, and Verruca, of which Phaeophyta and Bacteroides account for 60-90% of the alliance (8). It is an organism that coevolved with its human host and has more than 500 times as many genes (9). Evidence shows that intestinal microbiota is associated with obesity and obesity-related complications such as T2DM and non-alcoholic fatty liver disease (NAFLD) (10). Many early classic fecal transplantation experiments have shown that intestinal microbiota plays an important role in energy acquisition, VAT accumulation, and insulin resistance (11, 12).

Current treatment of T2DM includes lifestyle intervention, a balanced diet, proper exercise, and medication (13). In recent years, great progress has been made in the use of biguanides and sulfonylureas, but these hypoglycemic drugs still have some limitations due to adverse reactions (14–16). Therefore, attempts to find the treatment of metabolic diseases from natural products have been carried out one after another. Many studies have reported that BBR has obvious effects of reducing blood glucose and lipids, simultaneously anti-obesity and inflammation (17–19). Therefore, it is considered to be one of the most promising natural drugs for the treatment of T2DM. The purpose of this review is to clarify the relationship between T2DM, intestinal microbiota, and BBR, and to deepen the understanding of the mechanism by which BBR plays a role in the treatment of metabolic diseases. It may be helpful for future clinical use of BBR monomers or related formulations to manage T2DM and its complications by regulating intestinal microbiota.

## 2 Effect of Intestinal environment changes on T2DM

### 2.1 Changes in the richness of intestinal microbiota

The composition of intestinal microbiota is determined by the complex interaction of host heredity, diet, (congenital) immune factors, intestinal environment, and interspecific competition (20). The composition of intestinal microbiota is unique to everyone (21). Due to the high acidity and oxygen content of the stomach and duodenum, the microbial composition changes along the gastrointestinal tract (21). The stomach and small intestine are rich in *Firmicutes* (*Lactobacillaceae*) and *Proteobacteria* (*Enterobacteriaceae*), whereas the large intestine shows a higher portion of *Bacteroidetes* (*Bacteroidaceae*, Prevotellaceae, and *Rikenellaceae*) and *Firmicutes* (*Lachnospiraceae* and *Ruminococcaceae*) *(* 21). The proximal intestinal microbiota produces metabolites such as short-chain fatty acids (SCFAs) and succinic acid by fermenting dietary fiber, which can prevent obesity by increasing energy consumption, promoting anorexia hormone production, slowing gastrointestinal movement, and improving appetite regulation. The distal intestinal microbiota mainly degrades peptides and proteins to produce ammonia, phenols, and branched chain fatty acids by hydrolysis and fermentation, which are harmful to the intestinal and metabolic health of the host (22). In healthy individuals, the composition of intestinal microbiota is abundant, while the diversity of obese and T2DM patients is reduced (23). Two large meta-genomes in China and Europe were used to study the structural characteristics of intestinal microbiota in T2DM patients and healthy people (24–26). It was found that the bacteria rich in T2DM patients were mainly conditional pathogenic bacteria, such as *Escherichia coli*, *Clostridium*, and *Carbella*, etc., while the richness of SCFAs-producing bacteria such as *Enterobacter*, *Clostridium, SS3/4*, *Pseudomonas* and *Rosmarinus* decreased (25).

#### 2.1.1 *Akkermansia muciniphila*

Among many bacteria, *Akkermansia muciniphila* (*A.muciniphila*) has been proposed as a new marker of intestinal health because it is a key bacteria at the mucosal interface between the lumen and host cells and plays a very important role in the regulation of a series of metabolic diseases. *A.muciniphila* is a kind of mucus-degrading bacteria, which exists in the mucus layer and can stimulate the production of mucin in the host, thus strengthening the integrity of the epithelial layer. Studies have shown that *A.muciniphila* abundance decreases in obese and T2DM mice, while feeding normalizes *A.muciniphila* can reverse metabolic disorders caused by a high-fat diet (27). In a randomized, double-blind, placebo-controlled, single-center prospective clinical study, daily oral administration of 10^10^ A*.muciniphila*, both alive and pasteurized for three months, was safe and well tolerated. Compared with placebo, pasteurization *A.muciniphila* increased insulin sensitivity, and reduced insulinemia and plasma total cholesterol (28). Amuc1100 is a specific protein isolated from the outer membrane of *A.muciniphila*, which interacts with Toll-like receptor (TLR)2 to play an immunomodulatory role *in vivo* and *in vitro*, and improves the intestinal barrier function (29). Reduced *A.muciniphila* can be considered as a biomarker of individuals with prediabetes (30) and can be used for early diagnosis of T2DM before the clinical attack, which will help to promote microbial-mediated early intervention (31).

### 2.2 The changes of metabolites derived from intestinal microbiota

#### 2.2.1 SCFAs

*Butyrivibrio, Bifidobacterium bifidum, Megasphaera*, and *Prevotella* can produce SCFAs through the digestion of fibrous polysaccharides, including acetate, propionate, butyrate, etc. (32). SCFAs are an important energy source of colon cells and are related to the improvement of heat production and calorie intake (32). SCFAs play a key role in the homeostasis of glucose metabolism by reducing the oxidative stress of β-cell, increasing insulin release, and reducing the expression of proinflammatory cytokines and anti-lipolysis (33–36). In addition, SCFAs play another potential role in the treatment of T2DM through intestinal gluconeogenesis (IGN). Compared with hepatic gluconeogenesis, IGN accounts for only about 20-25% of total endogenous glucose production during fasting (37), and it may be related to the improvement of glucose homeostasis and the reduction of T2DM risk (38). The glucose produced by the intestine is sent to the portal vein, and the peripheral nervous system in the wall of the portal vein can sense glucose and send signals to the brain to regulate energy and glucose metabolism (37). Propionate and butyrate are typical representatives of SCFAs. Propionate can be used as the substrate of IGN to activate the expression of the IGN gene (free fatty acid receptor FFAR3) through the intracerebral neural circuit, and butyrate can directly stimulate the expression of the IGN gene in intestinal epithelial mucosa through the increase of intracellular cAMP (39). Importantly, SCFAs can also bind to FFAR2 and induce the release of cytoplasmic Ca^2+^ from intestinal epithelial L cells, which in turn promotes the synthesis and secretion of peptide (P)YY and glucagon-like peptide (GLP-1) (40). The release of (P)YY and GLP-1 is essential for pancreatic function, insulin secretion, and appetite regulation (35, 41, 42). The change in the production of SCFAs is also capable of modifying skeletal muscle metabolism and function. SCFAs have been shown to influence lipid, carbohydrate, and protein metabolism in skeletal muscle tissues both *in vitro* and *in vivo*. Furthermore, SCFAs have the potential to increase skeletal muscle mass retention, blood flow, and insulin sensitivity, and to preserve an oxidative phenotype. The activation of adenosine monophosphate-activated protein kinase (AMPK), peroxisome proliferator-activated receptor-δ (PPAR-δ), peroxisome proliferator-activated receptor-1α coactivator (PGC-1α), and the inhibition of histone deacetylases (HDACs) are likely key mechanisms through which SCFAs induce these changes to skeletal muscle (43, 44). In addition, SCFAs stimulate oxidative metabolism in the liver and VAT *via* activating AMPK to induce a reduction in body weight (45). SCFAs-producing bacteria (*Butyrivibrio, Bifidobacterium bifidum, Megasphaera*, and *Prevotella*) decreased significantly during T2DM, so the protective effect of SCFAs decreased accordingly (27).

#### 2.2.2 TMAO

*Clostridium XIVa strains, Eubacterium* sp. *strain AB3007*, and *Escherichia coli* can produce trimethylamine (TMA) by decomposing phosphatidyl, L-carnitine and choline in food, which can be converted into trimethylamine oxide (TMAO) in the liver through liver enzyme Flavinemonooxygenase3 (FMO3) (46). TMAO induces atherosclerosis in mice, which is related to the incidence of human cardiovascular disease (47). However, in recent years, the relationship between TMAO and T2DM has also received widespread attention. A study based on 2694 participants showed that there was a positive correlation between plasma TMAO concentration and T2DM in the Chinese population (48). The protein kinase RNA-like ER kinase (PERK) is a receptor for TMAO. TMAO binds to PERK at physiologically relevant concentrations, selectively activates the PERK branch of the unfolded protein response, and induces the transcription factor Forkhead Box O1 (FoxO1) in the liver, which is a key driver of metabolic disease (49). FOXO1 function is required for the robust activation of gluconeogenic gene expression in hepatic cells to promote hyperglycemia (50).

#### 2.2.3 Bile acids

In addition to producing new metabolites, intestinal microbiota may also change the physical and chemical properties of endogenous metabolites. Bile acids (Bas) are the main degradation product of cholesterol, which are used to dissolve lipids and fat-soluble vitamins and play an important role in regulating host energy metabolism. Primary Bas are susceptible to being modified by intestinal microbiota all along the intestinal tract (such as specific strains of Clostridium from the large intestine) (51, 52). These modifications include deconjugation (the removal of amino acid residues) *via* bile salt hydrolase (BSH) activity and further metabolization *via* the removal of hydroxyl groups (ihydroxylation), oxidation (dehydrogenation), or epimerization (51, 52). This results in the formation of secondary Bas such as deoxycholic acid, lithocholic acid, and ursodeoxycholic acid (a secondary BA in humans, although a primary BA in rodents). This bacterial metabolism changes the bioavailability and bioactivities of Bas, and consequently their impact on the metabolic responses they are involved in (53). Secondary Bas can better bind to Takeda G protein-coupled receptor 5 (TGR5) and farnesoid X receptor (FXR). TGR5 is one of the most important receptors of Bas. It is expressed in the intestine and pancreas. Bas activating TGR5 can promote the secretion of GLP-1 by intestinal L cells (54). FXR is another key receptor of Bas, which is highly expressed in the liver, intestine, and kidney. Many studies have shown that activating FXR can improve hyperglycemia and hyperlipidemia by both suppressing hepatic gluconeogenesis *via* FXR/miR-22-3p/PI3K/AKT/FoxO1 pathway and promoting glycogen synthesis through FXR/miR-22-3p/PI3K/AKT/GSK3β pathway (55–59).

#### 2.2.4 BCAAs and AAAs

Circulating branched-chain amino acids (BCAAs) and aromatic amino acids (AAAs) are related to insulin resistance and T2DM in prospective cohorts recently (60, 61). For example, Isoleucine, leucine, valine, phenylalanine, and tyrosine are markers of the development of insulin resistance in young, normoglycemic adults, with the most pronounced associations for men (62). The serum metabolome of insulin-resistant individuals is characterized by increased levels of BCAAs and AAAs, which correlate with intestinal microbiota that has an enriched biosynthetic potential for these amino acids (63). *Prevotella copri* and *Bacteroides vulgatus* are identified as the main species driving the association between the biosynthesis of these amino acids and insulin resistance, which can aggravate glucose intolerance and augment circulating levels of these amino acids (64). BCAAs and AAAs are vital to glucose and protein metabolism, and the increase of these amino acid levels may indicate the onset of T2DM (60, 65).

### 2.3 The immune regulation of intestinal microbiota in T2DM

**Figure 1:**
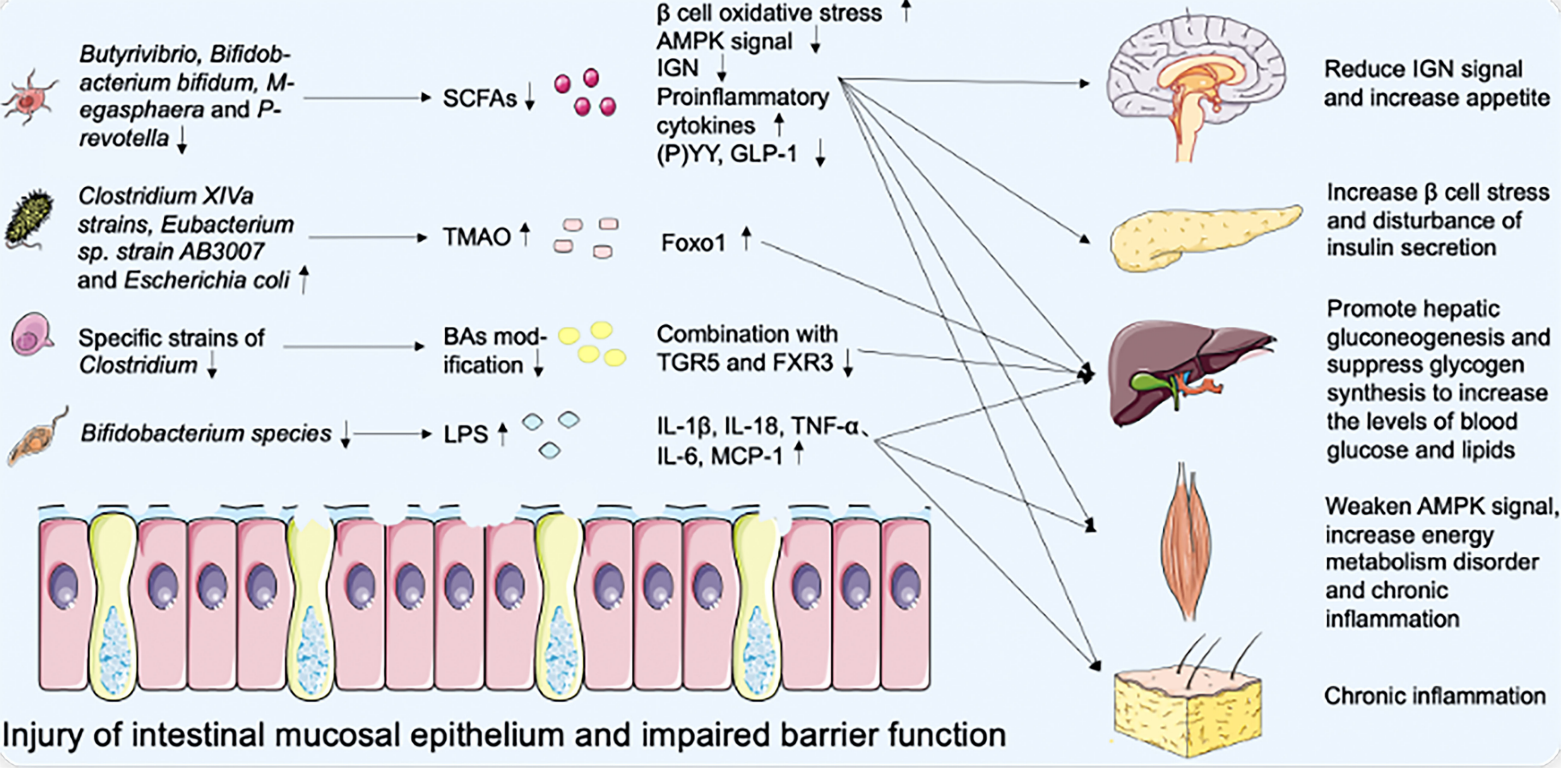
Effect of intestinal microbiota disorder on metabolic dysfunction of multiple organs.

In addition to metabolic interaction, changes in innate immune levels may also be important in the crosstalk between intestinal microbiota and host metabolism. The decrease of *Bifidobacterium species* and the destruction of the intestinal barrier promote the development of metabolic endotoxemia and increase the level of bacterial lipopolysaccharide (LPS) in plasma, which is a component released from the cell wall of Gram-negative bacteria (66). LPS is one of the pathogen-associated molecular patterns (PAMPs), which is recognized by pattern recognition receptors (PRRs), including Toll-like receptors (TLRs) and Nod-like receptors (NLRs) (67, 68). The interaction between PRRs and PAMPs induces the production of cytokines and interferon (IL-1β, IL-18, IL-6, TNF-α, and MCP-1), thus triggering the cascade of pro-inflammatory signals in the body’s peripheral tissue (VAT, liver, and muscle) (69). The inflammasome is the core component of innate immune response and is related to a variety of metabolic diseases (70). LPS can bind to TLR to activate the first signal of the inflammasome and damage-associated molecular patterns (DAMPs) can respond to activate the second signal (67). The inflammasome can promote the production of mature caspase-1, cut Pro-IL-1β and Pro-IL-18 to form mature IL-1β and IL-18, and then release them into extracellular tissue (71). T2DM is associated with increased levels of pro-inflammatory cytokines, chemokines, and inflammatory proteins (72, 73). Therefore, immune dysfunction is also nonnegligible in the pathogenesis of T2DM mediated by intestinal microbiota (74) (**Figure 1**).

In T2DM patients, the richness and quantity of bacteria changed, accompanied by intestinal mucosal epithelial injury and barrier dysfunction. The decrease of SCFAs secreted by *Butyrivibrio, Bifidobacterium bifidum, Megasphaera* and *Prevotella* leads to the increase of oxidative pressure of β cells and the secretion of proinflammatory cytokines, the downregulation of AMPK signal in the muscle, liver, and VAT, the decrease of P(YY), GLP-1 secretion and IGN proportion of intestinal epithelial L cells. Worsening blood glucose levels by targeting the nervous system, pancreatic β cells, liver, muscle, and VAT. At the same time, *Clostridium XIVa strains, Eubacterium* sp. *strain AB3007*, and *Escherichia coli* metabolize to produce TMAO, induce the expression of transcription factor Foxo1, and then promote hyperglycemia. Specific strains of Clostridium also reduce the dehydroxylation and hydrophobicity of BAs, which decreases the binding of BAs to TGR5 and FXR3 and increases the levels of blood glucose and lipids. In addition, the decrease of *Bifidobacterium species* and the destruction of the intestinal barrier promote the blood concentration of LPS, which induces the expression of cytokines IL-1β, IL-18, TNF-α, IL-6, MCP-1 and chronic inflammation of the body’s peripheral tissue.

## 3 Effects of BBR on intestinal microbiota and T2DM

Traditional Chinese medicine (TCM), also known as botanical medicine or phytomedicine, is a scientific and technological resource with treatment or other health benefits. BBR is an isoquinoline alkaloid derived from the stems and roots of Berberis species, including *B. Aristata, B. Darwinii, B. Petiolaris*, and *B. Vulgaris (* 75–77). BBR or herbs containing BBR have been used to treat intestinal infections, especially bacterial diarrhea in China for thousands of years (78). In recent years, its curative effect on T2DM patients has also been effectively proven (79–81) and it is widely believed to be an antidiabetic drug that regulates insulin signal transduction (82). Although BBR is a cationic alkaloid, its structure is not optimized for rapid intestinal absorption. The absolute bioavailability of BBR is much less than 1%, and the trace BBR absorbed from the intestine can be excreted from the ileal cavity through the action of P-glycoprotein (83). Due to the significant contradiction between low bioavailability and strong therapeutic effects, it is assumed that the regulation of intestinal microbiota may be one of the mechanisms of its anti-diabetic effect. Other studies have shown that intestinal microbiota may affect the absorptive activity of individual BBR. It is reported that intestinal microbiota can transform BBR to the absorbable form of dihydroberberine (dhBBR) in animals, and its intestinal absorption rate is 5 times higher than that of BBR. The absorbed dhBBR can then be oxidized back to BBR to enter the bloodstream (84). The above evidence suggests that the anti-T2DM mechanism of BBR is at least partly mediated by the regulation of the intestinal environment.

### 3.1 BBR changes the richness and quantity of intestinal microbiota

One of the most important functions of BBR is that it can change the composition of intestinal microbiota. A significant decrease in the total bacterial population was observed in rats treated with BBR. BBR has a broad antibacterial spectrum including opportunistic pathogens (*Staphylococcus, Streptococcus, Salmonella, Klebsiella*, and *Pseudomonas*), inducing death of harmful intestinal microbiota (*Escherichia coli*), enhancing the composition of beneficial bacteria (*Bifidobacterium adolescence, Lactobacillus acidophilus, and A.muciniphila*) *(* 85–89), and increasing the ratio of Phaeophyta to Bacteroides, etc. (90). All of these bacteria have a profound effect on blood glucose and lipids levels (91).

### 3.2 BBR improves SCFAs content and the intestinal barrier function

BBR can also increase the number of SCFAs-producing bacteria (*Blautia* and *Allobaculum*) in the intestinal tract (92). Meanwhile, levels of SCFAs are also significantly elevated after treatment with BBR (93). SCFAs work as a mediator between intestinal microbiota, they have the potential to improve glucose homeostasis and insulin sensitivity in patients with T2DM. In the setting of pancreatic dysfunction, they can regulate pancreatic insulin and glucagon secretion through GLP1 augmentation, meanwhile improving blood glucose levels by targeting the nervous system, pancreatic β cells, liver, muscle, and VAT (33). Apart from SCFAs-producing bacteria (93), the abundance of *A.muciniphila* in B6 mice induced by a high-fat diet was 19.1 times higher than that in the control group after BBR intervention, thus regulating tight junction protein and protecting the integrity of the intestinal barrier (94). BBR can increase the goblet cell number and villi length, and reverse the suppressed expressions of mucin, occludin, and zonula occludens-1 (ZO-1) to protect the intestinal barrier function (95).

### 3.3 BBR improves BAs and amino acids metabolism

Moreover, BBR can increase some beneficial bacteria with benzene sulfonyl hydrazine activity, for instance, *Bacteroides*, *Bifidobacterium, Lactobacillus*, and *Clostridium*, promote the decomposition of conjugated bile acids (CBA) and strengthen their excretion through the intestine. *Lactobacillus* converts primary BAs into secondary BAs through decarboxylation (96), and BBR can enhance the expression of FXR and TGR5, which is considered to be an agonist (97). The Phylum Firmicutes have higher BSH activity than the Phylum Bacteroidetes in the intestinal microbiota, and the latter is only active against taurine-conjugated BAs. BBR remarkably increased the *Firmicutes/Bacteroidetes* ratio and it is one of the mechanisms of its induced serum-free BAs increase and lipid-lowering effect (98). Overall, the mechanisms by which BBR alters the intestinal microbiota and improves metabolism are related to its choleretic effects.

In addition, the metabonomic analysis of colonic contents identified 55 different intestinal metabolites and showed that tyrosine, tryptophan, and phenylalanine in the BBR group decreased not only in the colonic contents but also in the serum, indicating that BBR can alleviate the symptoms of T2DM by reducing the contents of BCAAs and AAAs (99, 100).

### 3.4 BBR regulates intestinal immunity

BBR can significantly reduce the abundance of *Proteobacteria*, (such as *Desulfovibrio, and Enterobacter cloacae)*, and inhibit LPS production, as well as prevent serum LPS elevation, regulating intestinal permeability, attenuating insulin resistance and improving metabolic endotoxemia effectively (101). In diet-induced obese (DIO) mice, BBR decreased the levels of LPS and inflammatory mediators (IL-1, IL-6, TNF-α, and MCP-1), blocked the biosynthesis of TLR and NF-κB, improved intestinal and VAT inflammation, and promoted insulin signal transduction and glucose metabolism (102). By activating AMPK activity, BBR inhibited the production of IFN- γ, and Il-17A by lamina propria CD4+T cells *in vivo* and vitro (102, 103). At the same time, the expression of immune-related genes (including *Nfkb1, Stat1*, and *Ifnrg1*) in islets of the BBR group decreased significantly (93). These suggested that the anti-diabetes/obesity potential of BBR is related to its anti-inflammatory effect to some extent.

### 3.5 BBR promotes intestinal GLP-1 secretion

**Figure 2:**
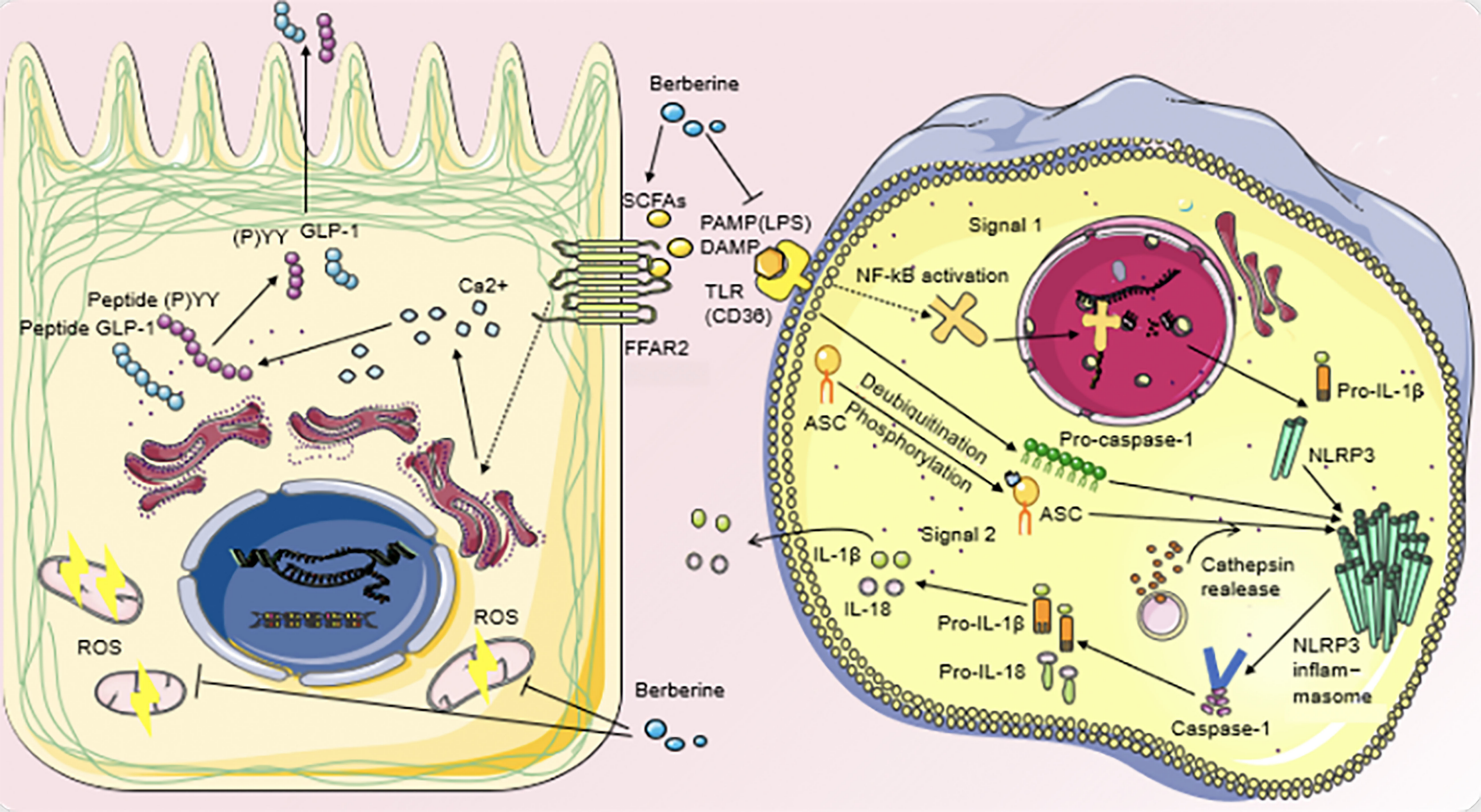
Regulatory mechanisms of BBR on metabolic disturbance.

Studies have shown that BBR decreases the variousness of intestinal microbiota, increases the proportion of *Bacteroidetes* and *Firmicutes*, further increases the number of L-cells in the proximal colon, and the expression of serum GLP-1, GLP-2 (95, 104, 105). BBR treatment can elevate plasma GLP-1 and orexin-a, and upregulate hypothalamic GLP-1 receptor expression, which has beneficial effects on various metabolic disorders such as insulin resistance and obesity, thereby inducing regulation of the gut-brain axis of the microbiota (106). It was observed that the decrease of GLP-1 was accompanied by the increase of mitochondrial stress response in the colonic cells of DIO mice. BBR administration not only restored GLP-1, but also relieved mitochondrial stress pressure, down-regulated intestinal transport speed and appetite, and improved energy metabolism in DIO mice (90) (**Figure 2**).

BBR can increase the number of SCFAs-producing bacteria and the production of SCFAs in T2DM patients. Its action on FFAR2 can release intracellular Ca^2+^ from intestinal epithelial L cells, activate the synthesis and secretion of GLP-1 and peptide (P)YY, and reduce the level of oxidative stress in L cell mitochondria and ROS production. LPS can bind to TLR to activate the first signal of the inflammasome and DAMP can respond to activate the second signal. The inflammasome can promote the production of mature caspase-1, cut Pro-IL-1β and Pro-IL-18 to form mature IL-1β and IL-18, and then release them into extracellular cells. BBR inhibits the production of bacterial LPS, blocks the biosynthesis of TLR and NF-κB, and reduces the secretion of IL-1β and IL-18 pro-inflammatory cytokines, thus slowing down the level of inflammation in the body’s peripheral organs and tissues.

### 3.6 Synergistic effect of BBR and other drugs

Early studies have shown that BBR can increase the oral bioavailability of other drugs. When combined with metformin, it can reduce the degradation of metformin by human and rat intestinal microbiota (107). Compared with anti-T2DM single drug/monomer therapy, the compound prescription of TCM accords with the newly proposed principle of “multi-drug and multi-target”. The synergistic effect of TCM includes synergism and toxicity reduction (108, 109), and the mixed bioactive components in the compound can enhance the curative effect of each other (110–112). For example, BBR combined with stachyose can improve glucose metabolism and intestinal microbiota disorder by regulating microRNA and gene expression in the colon of diabetic rats (113). The compatibility of *Rhizoma Coptidis* with *Cinnamon* improves T2DM and diabetic nephropathy more effectively by affecting the pharmacokinetics of BBR (114, 115). In short, these results show that BBR can further play a variety of health promotion effects through synergism.

### 3.7 Clinical trials of BBR in the treatment of hypometabolic disorders

We searched for registered clinical trials investigating the efficacy of BBR in the treatment of hypometabolic disorders. There were numerous and most of the published results reported a positive efficacy of BBR. (**Table 1**). However, mild diarrhea caused by BBR under clinical conditions can also happen and it is associated with intestinal microbiota disorder, such as the increase in the abundance of *Porphyromonas* and *Prevodiaceae*, as well as *Parasitobacteria*, *Prevodiaceae UCG-001* and *NK3B31* groups (116). But overall, the gastrointestinal reaction of BBR is mild and transient. Compared with its overwhelming health benefits, the side effects of BBR are sporadic. Further long-term toxicity studies on the low toxicity and side effects of BBR are still needed to prove BBR’s safety (80).

**Table 1:** Clinical trials of BBR registered at ClinicalTrials.gov in the treatment of glucose metabolic disorder.

Disease	Status	Phase I/II/III/IV	Number of Patients	Intervention measures	Dose	Duration	Start date	Identifier
Prediabetes	Completed	III	300	BBR/bifidobacterium/BBR, bifidobacterium/placebo	0.5 g, bid	16 weeks	October 2015	NCT03330184
Prediabetes	Active, not recruiting	IV	28	BBR/metformin	0.5 g, tid	14 weeks	March 2016	NCT03029390
Hyperglycemia	Unknown	IV	200	BBR, Insulin/Insulin	0.5 g, bid	8 days	July 2016	NCT02806999
Glucose Metabolism Disorders	Recruiting	–	50	BBR/placebo	0.55 g, bid	8 weeks	March 2020	NCT05031715
DM	Completed	IV	800	BBR/placebo	–	2 days	July 2016	NCT02808351
DM	Completed	I	–	BBR/placebo	–	–	October 2019	NCT03972215
T2DM	Completed	I, II	70	BBR/metformin	–	13 weeks	January 2004	NCT00425009
T2DM	–	III	400	BBR, probiotics/placebo, probiotics/BBR, placebo/placebo	0.6 g, bid	3 months	August 2016	NCT02861261
T2DM with Dyslipidemia	Completed	III	116	BBR/placebo	1.0 g/day	3 months	April 2005	NCT00462046
Metabolic Syndrome	Recruiting	–	40	BBR/placebo	1.5 g/day	–	August 2019	NCT03976336
Metabolic Syndrome	Not yet recruiting	IV	5200	BBR/Healthy lifestyle intervention	0.5 g, bid	3 years	December 2021	NCT05105321
Metabolic Syndrome	Not yet recruiting	III	40	BBR/placebo	0.5 g, tid	6 months	April 2021	NCT04860063

## 4. BBR efficacy in improving other metabolic diseases

### 4.1 Obesity

Compared with the model group, BBR can restore the relative level of *Bifidobacterium*, the ratio of Bacteroides to Phaeophyta (117), and the abundance of *A.muciniphila* (118). It can also increase the number of SCFAs-producing bacteria (*Isobacteria, Bacteroides, Brucella, Casein*, and *Bacillus)* in obese rats, and the concentration of total SCFAs (acetic acids and propionic acids) (119). In addition, oral BBR increases the secretion of GLP-1 in intestinal L cells, upregulates the levels of GLP-1 receptor, neuropeptide Y, and orexin An in the brain, improves the ultrastructure of the hypothalamus (106) and increases the expression of fasting-induced adipose factors in VAT (120). It can effectively reduce body weight and plasma lipid levels by regulating the microbiota-gut-brain axis.

### 4.2 Hyperlipidemia

A 3-month clinical study has shown that the effective cholesterol-lowering effect of BBR was closely related to the baseline levels of *Alistipes* and *Blautia* bacteria (121). BBR treatment led to the enrichment of beneficial bacteria (*Bacteroides and Blautia*) and the reduction of *Escherichia coli* (122), correcting the composition of intestinal microbiota and fungi to play a role of anti-hyperlipidemia (123). At the same time, the effect of BBR as a lipid reducer has been also proven to be related to Bas conversion and ileal FXR signal pathway. Animal metabonomic analysis showed that BBR treatment increased the levels of pyruvate, 5-hydroxytryptamine, ketogenic, and glycogen amino acids in serum, pyridoxine, and 4-pyridoxic acids in urine. Meanwhile, decreasing taurine and methionine in the liver, and deoxycholate and lithocholic in feces. Indicating that the changes in intestinal microbial metabolites can also affect the level of blood lipids (124, 125).

### 4.3 NAFLD

The level of hepatic BAs transporter is down-regulated due to liver inflammation, which slows down the enterohepatic circulation and promotes the increase of CBA in the serum and liver of patients with NAFLD. High levels of CBA activate sphingosine 1-phosphate receptor 2 (S1PR2) to activate pro-inflammatory and fibrosis pathways, which promotes the progression of non-alcoholic steatohepatitis (NASH). BBR as an anti-inflammatory compound combined with BAs receptor agonist can delay the progression of NASH and improve BAs metabolism by inhibiting microorganisms related to BSH activity, such as *Clostridium XIVa* and *IV* (124, 126, 127). A study explored the potential mechanism of BBR involved in the intestinal microbiota-immune system axis against NAFLD. It is worth noting that BBR activated an immunosuppressive population in the liver of mice to reduce alcoholic liver injury, which is defined as a granulocytic myeloid-derived suppressor cell (G-MDSC)-like population, and correspondingly reduced cytotoxic T cells and activated IL6/STAT3 signal transduction. These protective effects were eliminated after the consumption of intestinal microbiota, indicating that intestinal microbiota may be involved in mediating the expansion of the G-MDSC population (126, 128). In addition, BBR significantly restored the relative abundance of *Bifidobacterium*, *A.muciniphila*, and the ratio of Bacteroides to Phaeophyta, decreased the levels of serum LPS and several inflammatory cytokines (IL-1, IL-6, and TNF-α), improved the intestinal barrier dysfunction and prevented intestinal toxic substances from entering the liver through the hepatic portal system (117, 129).

## 5 Summary and prospect

BBR has multiple systemic activities on metabolism-related chronic diseases, including antioxidant/anti-inflammatory effects and changes in intestinal microbiota composition and metabolism. BBR works through the drug cloud (dCloud) mechanism. Unlike drug target effects, dCloud is defined as a set of terminal molecular events induced by drugs or related metabolites and the network connections between them. The therapeutic effect of BBR is the result of its dCloud effect on symptoms/signs and the root causes of the disease, which can explain why BBR plays a role in a variety of metabolism-related diseases.

After oral administration, BBR interacts with the intestinal microbiota. BBR can regulate the composition of intestinal microbiota and its metabolites, and intestinal microbiota can also transform BBR into dhBBR. In this process, two types of metabolites can be produced, including intestinal microbiota metabolites (from food and host) and secondary compounds of BBR. Understanding the two-way interaction between BBR and intestinal microbiota is helpful for us to apply BBR in pre-clinical and clinical interventions. However, due to the low bioavailability and natural yield of BBR, the future potential of bioactive natural products for the treatment of diabetes will be based on a structural modification to obtain safer, higher bioavailability and patentable compound molecules. And alternative replenishment methods that rely on biotechnology production and chemical synthesis should be developed to deal with the problem that natural product replenishment is difficult to meet the huge commercial market demand.

This paper introduces the pathogenesis of T2DM caused by intestinal microbiota disorder, meanwhile, focusing on the effective and important active ingredient BBR to treat T2DM by targeting intestinal microbiota. It may be a promising way to control T2DM because it can relieve T2DM through different mechanisms and ways of action, including antimicrobial and anti-inflammatory, protecting the intestinal barrier function, improving microbial metabolism, stabilizing intestinal hormones, and so on. In addition, the clinical trials of registered BBR for the treatment of glucose metabolism disorders are summarized, which is helpful for us to understand the current research and development of BBR and provide a basis for its clinical application in the future. The mechanism of BBR in treating other metabolic diseases (including obesity, hyperlipidemia, and NAFLD) through intestinal microbiota is also brought to notice. However, there is still a long way to go to explain the mechanism of BBR against metabolic diseases and its potential side effects, especially in the context of wide differences in the composition of intestinal microbiota among individuals. We should continue to strengthen the understanding of the common signal transduction mechanism in different bacteria to achieve standardized treatment by targeting common molecules or signaling pathways, and bear in mind the ultimate goal of translating knowledge into practice.

## Author contributions

QH and DW conceived the paper. QH, DW, and FL wrote the article. DW, HD, MG, YG, and QX revised the figures and reviewed the article. All authors reviewed and approved the final version of the manuscript.

## Funding

This study was supported by the National Natural Science Foundation of China, (Grant NO.82274470 and NO.81974567).

## Conflict of interest

The authors declare that the research was conducted in the absence of any commercial or financial relationships that could be construed as a potential conflict of interest.

## Publisher’s note

All claims expressed in this article are solely those of the authors and do not necessarily represent those of their affiliated organizations, or those of the publisher, the editors and the reviewers. Any product that may be evaluated in this article, or claim that may be made by its manufacturer, is not guaranteed or endorsed by the publisher.
